# Two Lectures on Water

**Published:** 1850-04

**Authors:** F. H. Gordon


### WESTERN JOURNAL

OF

### MEDICINE AND SURGERY.

APRIL, 1850.

Art. I.—*Two Lectures on Water.* Delivered to his Class, in Lebanon, Tennessee, by F. H. Gordon, M. D.

During our course, reference has been frequently made to water as a remedial agent, and it is so important an agent in the healing art, as to induce me to call your special attention to its consideration. Its use is coeval with medicine, and its modes of application numerous. Much more of its virtues is known among practitioners, than you will find in books—even enough, were it collected, to put to shame that humbug, based on one truth, called hydropathy. Your time will, therefere, be well spent in studying this important remedy. Water, as a hygienic drink,and as a solvent of other remedies, you find well discussed in your text books. Pharmacy could not do without it; in keeping the proximate elements of the blood in a state of solution, it is of paramount importance; but its other properties and uses require a more careful consideration from us, because they are more important to the practitioner.

As an *internal* remedy, its properties *vary according to its temperature*. At all temperatures which can be tolerated by the stomach, it increases the pulmonary, cutaneous, and renal secretions. When taken into the stomach, it soon finds its way into the circulation, through the radicals of the gastric veins, and is eliminated by the skin, kidneys and lungs. But if water be swallowed hot, say at ninety-eight degrees Fahrenheit, or upward, it stimulates and accelerates the circulation, and acts as a powerful diaphoretic. The perspiration will be in proportion to the elevation of temperature, and the amount swallowed in a given time. Cold (thirty-two to sixty degrees Fahrenheit) water swallowed, acts as a diuretic and antiphlogistic or sedative to an inflamed stomach. But if drunk tepid, (sixty to eighty degrees Fahrenheit) it is emetic. Thus you may cause a patient to urinate, sweat, or vomit, or to cease vomiting, according to the temperature of the water he drinks. A small amount of very warm or very cold water will generally relieve nausea; but if drunk largely, then it is emetic, diuretic, or diaphoretic, according to its temperature, as just stated.—Bearing these facts in mind, you will be able to explain why a thousand and one remedies have popularity for these virtues. I allude not alone to the opinions of the populace, but to those of the profession also.

Many remedies of high repute, would doubtless cease to be valued, were they administered without water. In this respect, our materia medica would be much impoverished. The physician orders a decoction of some foreign or indigenous plant; the urine flows freely, and he therefore sees with his own eyes that it is an excellent diuretic. He thus knows from experience that the books are correct. But let him order his remedy without water, and direct his patient to abstain from all drinks; then experience will, in regard to most of the reputable diuretics, teach him differently. Similar remarks are true of a very long list of diaphoretics. Numerous teas, infusions and decoctions, owe their reputation exclusively to the warm water swallowed during the administration of these supposed sudorifics. Let these supposed remedies be taken without water, and you will find a large majority of them incapable of exciting perspiration. Very warm water, freely swallowed, so powerfully promotes perspiration, that, in most cases, it needs no medication.

As an *external* remedy, water is still more valuable.—And the fact that the two extremes of temperature, denominated cold and warm, give it different virtues, makes it necessary to treat of its application, first, as cold water, and second, as warm water.

Cold water has been variously applied to the surface of the body, as by immersion, dash, stream, sponging, or wet linen and bladders of ice. Its effects vary with those modes of application in intensity, but not in kind.

1st. The first and most obvious effect of cold water applied to the surface, is reduction of its temperature, by the abstraction of caloric from the point of application. As a consequence, contraction of the part, and sluggish circulation soon result.

2d. When the vital phenomena of the part have been depressed for a time, reaction takes place; that is, the vital forces are roused, and all living phenomena increase.

3d. *Tonicity* is imparted directly to the point of application, and indirectly to the whole system.

But these effects vary greatly, according to the force of the application; and the force varies with the different modes of using it. Of course, the practitioner must let the object to be accomplished determine what mode of application should be adopted. When there is local inflammation to be reduced, by diminishing the flow of blood to the suffering point, the most appropriate modes of applying cold water are by wet linens, the sponge, bladders of ice, and the stream. The application must be moderate, gradual and long continued; but when the object is to impart tonicity or vigorous reaction, the water must be applied with speed and force enough to shock and rouse the latent energies; and here the dash and shower-bath are appropriate.

To apply these principles in practice; suppose you have a case of cerebritis, pneumonitis, or gastritis. Now, if you plunge the patient in cold water, or dash it on him, it is evident that you will cause violent reaction in an organ already excited too much, and thus aggravate the inflammation. But if you pour a gentle stream on the head, or apply bladders of ice to it, or sponge the chest or epigastrium, and keep up this practice, so as to reduce the augmented heat for a long time, it is plain, that you reduce the inflammation of the part, and lessen the tendency to reaction, in proportion to the time of the application.

On the contrary, suppose you have an emaciated and debilitated patient, suffering of some *functional* disease of the prima via, liver, spleen, bladder, or uterus; and suppose you protractedly sponge or pour cold water over the diseased organ. Your patient, for want of vital power, will ultimately collapse and die; or run into a chill, and, after a slow reaction, have high fever, with aggravation of the local lesion. But if you plunge the patient, or dash the coldest water forcibly on any part of the body, and let the skin be speedily wiped dry and wrapped up warmly, the dormant energies are roused, equilibrium of the circulation is produced, with vigorous reaction, and tonicity is imparted to the whole system.

Here may be a seeming inconsistency. We apply cold water slowly and protractedly, to subdue acute inflammation; but if the lesion be sub-acute, and especially if it be merely functional, then the dash is the remedy. The following physiological facts, will manifest the reason of the procedure.

1. The more vitality there is in the patient, or in the diseased part, the more speedy and vigorous will be the reaction from the use of cold applications.

2. The colder the water, and the more sudden and forcible the application, the more prompt and vigorous will be the reaction. According to these two principles, the promptitude and vigor of reaction, are in direct proportion to the amount of vitality in the patient, and the celerity with which heat is abstracted by the water.

3. A shock rouses every organ of the body, and gives increased action to the circulation and nervous fluid.

4. If there be organic lesion, or violent inflammation, a shock does violence to the diseased part, by forcing it to over action.

5. If by cold water, the vital movements be reduced below the natural standard, the reaction will exalt the vital phenomena as much above, as they had been depressed below the natural level.

It appears from these principles, that in cases of chronic disease with prostration, the slow application of cold water, would not be tolerated by the system of the patient; because the blood and vital fluid are both impoverished or diminished, and are therefore, incapable of keeping up the equilibrium of circulation, calorification and vitalization, in opposition to the continued abstraction of heat by cold water. On the contrary, in acute inflammation, where the vital fluid and blood are abundant, there is but little danger of depressing the vital movements too low, if prudence be observed. Some depression in the diseased organ is the desideratum, and there is a continual strong tendency to reaction. And where there is serious organic disease, cold water is inadmissible.

From the foregoing considerations, the application of cold water in its different modes, to the various cases, may be easily determined. The cases requiring the cold stream, sponging, or bladders of ice water are: fever with general heat of surface, arachnitis, gastritis, enteritis, strangulated hernia, epistaxis, and gonorrhea, &c. And the cold dash is applicable to uterine hemorrhage with prostration, narcotism, syncope, catarrh, dyspepsia, amenorrhea, chronic diarrhea, chlorosis, and all chronic diseases with general debility, provided there be no serious organic lesion.

Your attention will now be called to the external application of *warm water*. The modes of its application to the surface of the body are immersion, vapor bath, poultices, fomentation, and a stream. Warm water is still the same agent, and its *effects are the same in kind*, whether applied by one, or any of these modes. We have seen, that when swallowed it is a stimulant, emetic, diaphoretic, and in common with cold water, it is diuretic; but when applied to the surface it displays other properties.

1. It softens and relaxes the skin and muscles.

2. It promotes cutaneous perspiration and general absorption.

3. It acts as a sedative to the nervous system, and thus quiets or subdues irritation.

4. It diminishes arterial action and equalizes the circulation, and thus removes local congestion and inflammation.

Warm water is still the same agent, having these effects, whatever may be its mode of application. But its potency is regulated by three circumstances, viz.: the *length of time*, the *constancy* and *force* of its application. The longer the application is continued, the greater its force, and the more constant, the more powerful will be its effects. The value of each mode is easily determined by these conditions.

The vapor bath, was at one time highly estimated in this country. It is conducted by generating steam in some vessel, and conducting it around the patient under the bed clothes. This mode of applying warm water has now but few advocates, owing no doubt to one peculiarity, that is, by surrounding the surface with an atmosphere highly charged with moisture, it impedes, if it does not arrest all cutaneous transpiration. The interferance with this important function diminishes its value, if it does not hinder the other legitimate effects of water.

Fomentation is a mode of applying warm water, by dipping flannal cloths in it, and applying them to the surface. It is an old remedy, and is much used at this day. But it is the least efficient of all the modes; because there is but little force in the application, and the cloths cool so rapidly as to require frequent changing. Yet from its convenience, it answers a valuable purpose in many instances, where a more powerful effect is not demanded.

The poultice is a much more potent antiphlogistic, and in subduing local inflammation it is deservedly popular. Yet there are frequent failures in securing the good effects of this remedy, because of the injudicious manner of its application. The poultice, to do great service, must be *large, light, and frequently changed.* It must be large, because a small poultice cools too speedily; it must be light, because otherwise its weight would oppress the patient; and it must be frequently changed, to keep up a temperature as nearly uniform as possible. To accomplish all of these objects, wheat bran is the best material for a poultice, and should be managed in the following manner: Suppose you have a case of peritonitis, put ten quarts of wheat bran in an iron pot; gradually add water, and stir till the whole mass is *merely moist* or damp throughout. Now heat it and stir occasionally till it is quite hot. Put half of this in a bag not less than eighteen inches square, (a pillow case is a suitable bag for this purpose,) spread the bran out, and apply the poultice, as hot as the patient can bear it, to the surface of the abdomen. At the end of twenty minutes have the remainder of the bran ready in another bag, and apply it the instant the former poultice is removed. Return the cool bran of the former poultice to the pot, to be ready to be reapplied at the end of twenty minutes more; and thus continue to change the poultices every twenty minutes, so as to keep up a temperature as nearly uniform as possible. Poulticing in this way for four or five hours will do more in subduing inflammation than a little and badly managed one could in a week. In fact, a little poultice is the mere semblance of a remedy; but when all of the conditions here named are observed, the poultice is powerful. I have named wheat bran as the best material; but poultices are made of various substances, many of which are supposed to possess some peculiar virtues, apart from the water they contain. But I suppose that the remedial agency of all or nearly all poultices is due to the water alone, which they contain. The tansy, hot ashes, cedar leaves, yarrow, corn mush, and wheat bran are only the means of absorbing warm water, and holding it in contact with the surface. And we frequently see poultices medicated with oak ooze, vinegar, and other means, under the belief that these means have some agency in reducing the inflammation. Such medications of course do no injury, and I never object to them, but gratify the friends of the patient by allowing any such medication, provided the poultices are made large and hot, and are changed frequently; for my reliance is on the water in the poultice.

Bathing, by immersing the patient partially or generally in warm water, is a remedy of great power in equalising the circulation, and promoting perspiration. But when the object is to subdue local inflammation, this remedy is not appropriate it acts so powerfully upon the nervous system as not to be tolerated by most patients, more than from fifteen to thirty minutes. This is too short a time for any material reduction of the inflammatory action. The powerful constitutional impression of warm bathing is owing to the great extent of surface acted on by the water.

The stream is the most valuable form of applying warm water *for the cure of local inflammation.* I have been familiar with it for years. It had been used by surgeons to relax the muscles in reducing luxations; but I am not aware of its having been employed for the reduction of local inflammation before I introduced it into the practice. For this latter purpose I am acquainted with no remedy which can equal it in value. I do not wish to overrate it or to appear to exaggerate its powers, for this would prevent a proper estimate from being placed upon it. Yet, to those who have not tried the remedy, the whole truth will look like exaggeration. Cups, leeches, blisters, and the lancet, do not equal it. The stream is a mode of applying warm water which complies with all of the conditions requisite to make it efficacious. It is *more constant*, may be *longer continued*, and applied with *more force* than any other application of water. The stream may be conducted almost without variation of temperature or force for hours, and, falling *only upon the suffering point*, it does not prostrate the patient like the bath, and hence may be continued long enough to greatly subdue the local inflammation before there is much depression of the vital powers. The *force* of the stream is an item of peculiar importance, because it is incomparably greater than that of any other mode of using warm water. It is a well known fact, that *fluids in motion* manifest their peculiar powers in a much higher degree than when at rest. The sultry air of a summer's day, which almost melts us by its quiescence, may, when put in motion, make hail, and chill the body. Electricity, which gently pervades our bodies at all times and stimulates all of the vital movements without misrule, if disturbed in its equilibrium and transmitted through the organs, with its incalculable velocity, will produce instant disorganization and death. Caloric, which pervades all of the tissues, and without which the vital functions could not be performed for one moment, does no injury while latent or in gentle motion; but when a large amount passes in or out of any point of the surface in a short time, destruction is the instant result. In like manner, water at rest in contact with the body will abstract or impart caloric according to its temperature, in so gradual a manner as to cause no very striking results; but when a swift current of water strikes the surface, its effects are manifestly in proportion to its velocity. Hence the stream of warm water as far surpasses in virtue the vapor bath and fomenting cloths, as the gentle breeze which wafts a feather is transcended by the tornado which uproots the sturdy oak.

But this, like all other remedies, may lose more than half of its virtues by being badly applied; it is, therefore, important that some special directions be given for its employment. Your object is to cause a *constant stream to fall with force upon the suffering point.* You may accomplish all of these objects by pouring with two pitchers, alternately, so as never to stop the stream longer than is necessary to exchange an empty pitcher for a full one. Or two coffee-pots or tea-pots will answer the same purpose. But the syphon is far the best instrument. A leaden tube from two and a half to three feet long, or a bent tin tube, or two joints of cane mitred together, secured with twine and made air-tight, will answer the purpose. The outer leg of the syphon must be from two to four inches longer than the one placed in the water. The diameter of the instrument must be from one-eighth to three-eighths of an inch, according as the patient is prostrated or vigorous. To understand the application of this instrument, particularly on the trunk, suppose you wish to apply the stream in a case of peritonitis. Take everything from the bedstead down to the cords; fold the bed-clothes and place them on the cords so as to make two beds of them, of equal height, with an interval of two inches between them, about where the umbilicus of the patient will be when he is placed upon them. Now place the patient, supine, upon the beds, with his umbilicus over the interval and a pillow under his head. Two blankets must cover the patient above and below the umbilicus, so as to leave a space of four inches between them. Pass two silk handkerchiefs around the body of the patient, one just above and the other just below the umbilicus, with their ends hanging through the cords to conduct the water. Have the clothes of the patient well pulled up, so that they may not get wet. A tub is placed under the bed to catch the water. A plank rests on the head and foot board, on which a bucket of warm water is placed. One end of the syphon is put into the bucket and made steady by a weight tied to it, and the other end presents over the umbilicus of the patient. By applying the mouth to the outer end and sucking, the stream begins, and ought to be adjusted to make it fall upon the most tender point. It should fall five or six inches, from the end of the syphon to the skin of the patient, or as far as it can without making a spray. The water should be tempered in a separate vessel and poured in the one which feeds the syphon, so as to keep it full and cause the stream to be uniform in temperature and force. The stream should fall constantly in the same place, because its effects will thus be greater than if moved frequently. The length of time consumed in using the stream must vary from one to five hours, according to the strength of the patient. Mustard plasters on the hands and feet will enable him to stand it much longer than without them. The rule is to continue the stream till the patient's pulse becomes small and frequent; and the higher the temperature of the water the longer it can be tolerated without faltering of the pulse. What should be the temperature of the water? At the beginning it should be about ninety degrees, Fahrenheit, and gradually increased till it reddens the skin slightly; this will be between one hundred and one hundred and fifteen degrees, according to the excitability of the patient. It is usual for patients to enjoy the stream finely for the first hour or two, but afterwards they become restless and insist on moving. They must be carefully instructed never to move themselves, for their clothes and bed would get wet. By rolling up a towel and placing it under one shoulder and another under the hip of the same side, the patient will be made to rest for a while, and when he again becomes tired place the towels under the opposite hip and shoulder. In this way you may make him contented till the bathing is ended, and all means should be employed for this purpose, for the effects of the stream increases almost in fourfold proportion with the time. In fact the last hour appears to accumulate as much good as the three preceding ones.

The first effect of the stream is on the point where it falls, subduing the inflammation for a small space around. The reduction gradually extends to more remote parts, as may be proven by gentle pressure being gradually tolerated progressively outward from where the stream falls. And, in the case of peritonitis before us, the legs can be completely extended without pain.

The second effect is upon the muscular, circulatory, and nervous systems. General relaxation ensues; the pulse loses its hardness, becomes full and elastic, and, ultimately, is small and frequent, but retains its softness.—At first, the patient is soothed; all pains cease and he falls to sleep, but afterwards awakes and becomes restless and weary of his position. These constitutional effects are so gradual, that you may almost, and sometimes entirely, subdue the local inflammation, before the patient becomes so much depressed as to require the discontinuance of the remedy. This is the excellency of the stream —that it acts with power enough *upon the point of disease* to subdue the inflammation in part or entirely, with but temporary depression of the general energies. The general febrile symptoms moderate and disappear while the stream flows, with as much certainty as can be expected from the lancet, and yet without ultimate loss of strength. It thus often does away the necessity of bleeding, and may be used in cases of great prostration, where no one would think of using the lancet. To a considerable extent, the warm stream supercedes the painful practice of blistering, relieving the disease without it; and where it does not subdue the local lesion entirely, it always diminishes it greatly, if continued long enough, and being on the decline, the blister plaster may be at once applied, with full prospect of good effects. And to speak in general terms, in all cases of local inflammation, the patient gets well sooner by the aid of the stream, than he could possibly without it, and suffers incomparably less during his illness. Unlike bleeding, purging, blistering, and all other powerful means of subduing inflammation, the warm stream may be repeated as often as may be needed. After all that has been said, need it be urged that we have no remedy so potent in reducing local inflammation? Let it be well tried, in a variety of cases, and you will be astonished at its effects. To see so pleasant a remedy relieve the most intense agony, remove all soreness, subdue the fever, and restore the pulse almost to its normal characteristics, in a few hours, almost passes belief. And though you will not be fortunate enough to see this picture universally, yet you may often be gratified with the sight.

You ask, in what cases is the stream applicable? I reply, *in all cases where there is local inflammation*, no matter what may be the name of the disease, whether acute or chronic, the stream will do good, and the nearer it can be made to fall on the point of the most intense suffering the better. It is true, that its effect will be more striking in acute disease, but it will be serviceable in chronic also. In mild or slight cases, I do not always take the trouble of applying the stream, but whenever I find severe suffering, or the life of the patient in jeopardy, I do not hesitate to direct the stream upon the suffering point.

I have watched this remedy in its extensive application, for many years, and could adduce from memory and my notes, a very long list of cases, tending to show its value; but must be content to bring forward comparatively few of them taken promiscuously from the mass in my possession, to which I ask your patient attention.

1. *Luxation of the Shoulder.*—Eli Harris, of this county, æt. 40, had his right shoulder dislocated, forward under the clavicle, on the 12th September last. A few hours afterwards, a neighboring practitioner saw him, and for four or five hours employed the strength of several strong men, in fruitless efforts to reduce it. On the 13th, about twenty-six hours after the accident, I found the patient discouraged, and the parts very sore and swollen from the previous efforts at reduction. I ordered a stream of warm water, about ninety-six degrees Fahrenheit, to be poured upon the inflamed joint for two hours, and with the aid of one man reduced the luxation with but little pain to the patient.

2. *Chronic Hepatitis with Gastrodynia.*—Mrs. Springs, of this county, was in a state of extreme prostration, in 1846. For four years, continued medical aid had done nothing towards a cure, and had afforded but occasional relief of her extreme suffering. There was general tenderness of the whole abdomen; the liver was much enlarged, extending below the umbilicus, and quite hard.—I directed the bowels to be kept regular, with pills of aloes, rhubarb and soap; diet, animal food exclusively, and a stream of warm water to be poured four hours daily over the region of the liver. On the second day, the liver was found soft for two inches in diameter where the stream had fallen. The stream was made to fall on a new place every day, and continued to soften the liver where it fell, till at the end of three weeks, the whole organ was reduced to its normal state, and the gastrodynia had disappeared. No mercury was used to act on the liver, and the recovery of the patient, in some four months, was due almost exclusively to the stream.

3. *Ptyalism from Mercury.*—In 1846, Miss Miner, of Smith county, was severely salivated. Could not rest; she had several ulcers; the pain, heat, and swelling were very great. The warm stream was directed on the cheek for two hours daily, and a mouth wash of borate sodæ used. Each application of the stream subdued the fever, and gave entire comfort. The mouth was well in a few days.

4. *Gastritis Acute.*—I was called to George Clope, of Wilson county, September 18, 1844. Had autumnal fever. Tongue red and dry; pulse small, hard, and one hundred per minute; frequent vomiting, and great tenderness of the epigastrium. Bled twenty ounces, with some relief. Gave minute doses of calomel and Dover's powder, and directed wheat bran poultices to the whole abdomen.

20th. Patient decidedly worse. Poured the warm stream upon the epigastrium for four hours, and directed two grains quinine every two hours.

22d. So much improved that further treatment was unnecessary, and the patient was up in a few days.

5. *Enteritis Acute.*—John Davidson, of Smith county, in 1846, had miasmatic fever, with enteritis, involving the peritoneum. Could not bear the slightest pressure. The stream of warm water, directed upon the umbilicus four hours, and repeated the day after, relieved all distressing symptoms, and a blister completed the cure.

6. *Purulent Ophthalmia.*—In 1845 the Egyptian ophthalmia was endemic in this region. I. Kilzer had violent pain in both eyes, balls swollen, conjunctiva looked like clots of blood, complete intolerance of light. Bled him copiously, gave calomel and directed the warm stream to be poured alternately upon the eyes till both should be comfortable. The next day no improvement, suspected the stream had been badly conducted, and poured it myself one hour upon each eye. Left patient comfortable. The family repeated the stream twice more on the two following days, and he was well in a week.

7. *Cynanche Maligna.*—Sept., 1849, Tabitha Moore, of Smith county, æt. three years, had cynanche, suffocating respiration, tonsils swollen, and of a dark purple color, pulse one hundred and fifty and small. At 12 M., applied a solution of nit. silver, fifteen grs. to ℥j. of water, to the tonsils and fauces, and gave tart. emetic freely. No nausea and no improvement. Gave 10 gr. blue mass, and reddened the throat with turpentine liniment. Still no relief. Gave 6 grs. calomel with 1 gr. tart. emetic, and 2 gr. ipecac; no nausea; and the child grew worse in all respects. About 9 P. M., had Dr. Thompson called in consultation, and told the family the child would die within fifteen hours, but determined to use the warm stream. It was poured on the throat two hours. The child breathed much better; broken doses of ipecac., were then repeated frequently, and nausea and vomiting were easily produced, followed by rapid improvement. Ipecac discontinued in three hours after its commencement; the child slept till daylight, and was out of danger.

8. *Pneumonia.*—I. Bradford, of Smith county, aged forty-five, was attacked with pneumonia 2d of April, 1846. Saw him on the 5th: dyspnea, constant dry cough, pulse one hundred and ten; two thirds of left lung impervious to air, and flat sound on percussion. Directed tartar emetic, repeated so as not to excite vomiting, patient to be bled, *ad deliquium*, at 2 P. M., and the stream to be poured to the left of sternum during alternate four hours, till my return next day. April 6, twenty-four hours after first visit. Patient better, expectorates some rusty sputa, coughs less, breathes better, pulse ninety-five, some crepitation in left lung. Has taken tartar regularly, and the stream has been poured twelve hours out of the twenty-four; was not bled, because the neighborhood bleeder thought he had not fever enough. Directions same as yesterday, except that the stream is to be poured eight hours out of the next twenty four. April 7.—Patient looks bright, much improved, full expectoration of brick-dust sputa; coughs but little and without pain, skin moist, pulse eighty-seven, sleeps well, left lung not so dull, some crepitus throughout the inflamed lung. The tartar and bathing had been used as directed, but bleeding had been again omitted for the same reasons as before. A blister was ordered over left lung, tartar water continued one day longer and then left off. Patient recovered in a few days.

9. *Nephritis.*—Miss L. Tyree, æt. eighteen, was attacked October 9, 1844. Saw her on the 11th. Had constant fever, headache, some pain in left kidney, extending into the pelvis, frequent scanty micturition, bowels constipated. The abdominal aorta pulsated inordinately down to the emulgents, thence the strong pulsation was traced out to the left kidney, which itself pulsated violently in unison with the heart. The stream was poured four hours upon the region of the kidney, which relieved the pain and subdued the fever. Ten grs. calomel, and 5 grs. Dover's powder were given, and mild diuretics directed thrice a day. On the 16th, patient was well. This patient had two other attacks of the same sort during 1845, which were relieved by the same treatment.

10. *Puerperal Peritonitis.*—Mrs. Dowty, of Smith county, aged forty, a delicate woman, was delivered of a child 1st May, 1847. Saw her the 5th, extremely ill, pulse one hundred and forty, small and corded, thirst, nausea, jactitation, delirium; abdomen swollen, quite tender and painful, legs flexed, lochia suspended, diarrhea. Directed 10 grs. calomel, and 1½ grs. morphia to be divided into three portions, one to be given every three hours. Poured the stream on the hypogastrium, for four hours, while mustard was used on the hands and feet, and directed the same to be repeated the next day; wheat bran poultice over the abdomen in the interval. 7th.—Patient much relieved, has had two consistent bilious dejections, abdominal tenderness nearly removed, mind clear, lochia returned, pulse one hundred and ten. Directed 3 grs. quinine every three hours, and ½ gr. morphia at bedtime. 9th.—Improving, quinine continued, poultice of wheat bran to the abdomen. 11th.—Sits up a little, appetite good, directed aloetic pills at bed time, to be checked with Dover's powder and acetate of lead, if the purgation should be too free. 13th.—Cured.

11. *Cystitis.*—Mrs. M., of Smith county, October, 1839, had acute inflammation of the urinary bladder, frequent micturition with burning pain in the urethra, and spasm of the bladder, whenever urine was discharged; tenderness of the hypogastrium, pulse frequent and small, profuse perspiration from pain. The lancet and hip bathing were used to some advantage, but the symptoms returned with violence. The warm stream was poured just above the pubis for five hours, with perfect relief of all pain. Mild diuretics and spare diet completed the cure without further suffering of consequence.

12. *Paraphymosis.*—A son of P. Smith, of Wilson county, æt. five years, was seen October 9th, 1846. The part of the prepuce forming the ligature completely buried, the glans swollen to one and a quarter inches in diameter and dark colored; micturition impossible. The warm stream poured on the swollen glans, for one and a half hours, made reduction easy, by compression between the palms of the hands.

13. *Orchitis.*—Ned, a negro man of J. Bradford's, æt. forty-five, had orchitis, July 1st, 1844, which yielded to ordinary treatment. August 20th, visited his wife five miles distant. Returned home with swollen and painful testicle. Was neglected, because of his imprudence, till 25th, when I found him in great torture, pulse one hundred and forty and irregular, clammy perspiration, extremities cold, the pain in the testicle extended to the loins. Directed 12 grs. calomel and 6 grs. Dover's powder; the warm stream to be poured on the testicle for six hours. After the use of the stream the patient slept soundly. 26th.—Comfortable; testicle a little softened, pulse one hundred, calomel had operated well. Directed strong iodine ointment to be freely rubbed daily on the testicle, and a suspensory bandage, recumbency, bowels to be kept regular with aloetic pills. Patient recovered in three weeks, but the epedidymis suppurated and was lanced.

14. *Croup.*—A daughter of J. Mitchell, æt. two years, was attacked 1st April, 1846, in the evening, got better before day; was taken very ill the evening of the 2d. Saw her at 9 P. M. Croaking cough, great distress in breathing. Gave tart. emetic and ipecac in broken doses, repeated often. No nausea. Reddened the throat with turpentine liniment, and immersed the child for half an hour up to the chin in warm water; gave 10 grs. calomel and 5 grs. ipecac. The child grew worse. After trying these, and other means, in vain for the whole night, the case appeared to be hopeless. About 9 A. M., on the 3d, directed the stream of warm water on the larynx for three hours, mean time gave ipecac every ten or fifteen minutes; the child vomited several times while under the influence of the stream, and was easily kept sick for several hours afterwards. Recovery complete on the 4th.

15. *Inflamed Mammary Gland.*—1843, Mrs. A. Garret, of Smith county, æt. forty-one, had great swelling, pain and heat of the right breast. Directed the stream of warm water to be poured for three hours on the tumor, and spts. mindereri to be taken, at intervals, for five hours so as to keep up constant nausea. The symptoms abated, and the patient recovered without further suffering.

16. *Torticollis.*—In 1846, I. Moore, of Smith county, slept near a window, from which a cold current of air blew on him. I found his head drawn down to the right shoulder; pain severe, when moved. The stream was directed on the right sterno-mastoid muscle for five hours. Patient could move his head normally, and was cured.

17. *Chronic Enteritis, with Induration.*—July 30, 1846, saw E. Atwood, of Smith county, æt. about 30. In the forepart of June, while overheated in the harvest field, had indulged freely in drinking very cold water. Was soon attacked with gastro-enteritis. Was confined to bed, under the regular treatment of a neighboring practitioner up to the 30th of July, when he became my patient. I found his tongue very red; bowels constipated; whole abdomen hard, and tender on pressure; whenever he was raised up, he complained of a heavy dragging weight of the bowels, as if they would tear, and the patient became sick and faint; he cannot lie on either side. Directed half a teaspoonful of powdered cubebs, nutmeg or ginger at each meal, carbonate and sulphate of magnesia, in equal portions, to be taken four times daily, in such quantities as would move the bowels gently; the warm stream to be poured four hours daily, near the umbilicus, where the bowels felt the hardest. In ten days the bowels became somewhat soft at this point, and blister plasters, of the size of a dollar, were made to migrate around the umbilicus, so as continually to draw a fresh blister. Meantime the stream was directed upon other parts of the abdomen, every third or fourth day, for several weeks. Patient to take sarsaparilla bitters. He slowly recovered, and was well about the 30th of November.

18. *Simple Metritis.*—This disease was endemic here, in the year 1844. I treated more than fifty cases; they were generally attended with uterine hemorrhage. The warm stream above the pubis, and warm water thrown up the vagina with a large syringe or syphon, were much used, and always with benefit, and sometimes prompt relief.

19. *Hypertrophy of the Uterus, with Procidentia.*—Mrs. O., of this county, æt. 41, had suffered from dysmenorrhea for several years, sometimes it amounted to menorrhagia, or even flooding. She had been under a judicious practitioner for two or three years, with occasional benefit. In 1846, I found her with globus, palpitations, cold feet, intermitting pulse, leucorrhea and amenorrhea; the womb hard to the touch and painful, about three inches in its transverse diameter, and resting with its mouth upon the perineum, causing dragging weight in the pelvis, pain in the loins, and numbness in the lower limbs. Directed aloetic pills to regulate the bowels, quinine every morning, a teaspoonful of pulverized cubebs before each meal; warm water to be *abundantly* thrown up the vagina daily, with a large self-operating syringe. In two months the patient was better. Directed the stream to be conducted up to the womb, with a syphon, for two hours every third day. This remedy was used but twice, and with so much benefit, that I saw my patient, soon after, at a camp meeting, five miles from home. The syphon was irregularly used for a month afterwards. Menstruation became regular and natural, all symptoms disappeared and health was restored.

20. *Vaginitis.*—In 1846, a negro girl, belonging to John Hause, of Smith county, had autumnal fever.—When she had been recovering for several days, it was ascertained that the entire mucous surface of the vagina and labia were discharging pus freely, and that, from incontinence of urine, the inner sides of the thighs were excoriated. The suffering was severe, constitutional disturbançe considerable, and the patient could not sleep.—Directed the warm stream to be conducted into the vagina, with a syphon, for two hours daily, the thighs and external genitals to be washed and oiled frequently. Patient slept several hours after the first use of the syphon, and was well in four days.

21. *Dysmenorrhea.*—Mrs. B., æt. 25, the mother of one child, six years old, when I saw her in 1844. She had suffered painful menstruation for four years and had been under treatment for three years. Every six or eight weeks she was supposed to suffer from an abortion, followed by profuse hemorrhage. I attended her in one of these supposed abortions. A transparent shut sack was discharged, about two inches in diameter, and filled with a transparent serous fluid. The pains were severe, and the subsequent hemorrhage prostrating. Directed an eight-ounce self-operating syringe to be injected twenty times full of warm water up to the womb every day.—Tarter emetic ointment rubbed on the spine, and the usual constitutional remedies. In about three months she began to menstruate naturally, and was delivered of a healthy child in 1845.

22. *Leucorrhea.*—This disease can not resist a free use of warm water, thrown up the vagina daily. But the common little cylindrical female syringe is useless. Its use may cleanse the surface, but this is all it can do. It is difficult to introduce into the vagina, and does but little good when introduced. This instrument ought to be abandoned by the profession, and the self-operating syringe or the syphon substituted. It takes much water to cure inflammation. I have often known patients to fumble for weeks with the clumsy instrument I have mentioned, irritating the inflamed vagina as much as the process benefitted it; and then I have known the little instrument laid aside and the large self-operating syringe substituted, when the discharge would cease in a short time.

24. *Amenorrhea.*—I have often known the menstrual flux to follow immediately after an abundant injection of tepid water into the vagina. But usually weeks or months are required to produce the effect. Yet you ought to bear in mind, that this suspension of the secretion is owing to some grade of inflammation in the uterus, and the more water you direct to the womb, the sooner you will remove the cause and restore the function. And let it be here impressed, that *in all cases the water thrown into the vagina must be merely tepid,* (about seventy degrees Fahrenheit); for if as high as blood heat or higher, it will cause a distressing vertigo.

25. *Crural Phlebitis, (Phlegmasia Dolens.)*—12th October, 1846, I saw Maria, æt. 38, a servant of D. Palmer, of Smith county. Left lower extremity considerably swollen, as well as the labium of that side, very tender to the touch, particularly in the course of the veins; she could not bear the weight of the bed clothes, or extend the limb. Gave twelve grains blue mass, directed the warm stream to be poured three hours upon the femoral vein, just below the crural arch, to be repeated daily till relieved; and the limb to be bandaged just after each use of the stream. The first application gave striking relief.

13th. Patient much improved; limb not so tender, nor so tense; can be extended. The stream was used as before, and the bandage immediately applied from the toes to the hip. From this moment, the patient ceased to suffer, and needed no further treatment, except to reapply the bandage, so as to follow up the shrinking of the limb.

In a week from the beginning of the treatment, the patient was walking about cured. During the same year, four other cases of this affection were treated with the stream of water, with results equally striking and prompt. This treatment was suggested by the pathology of the disease, as demonstrated by Robert Lee; and if the treatment is used early, it appears to be competent to cure the disease. How it would succeed in the advanced stage of the disease, I have had no opportunity to ascertain.

The foregoing cases, treated with the warm stream of water, must be sufficient to show its value clearly. Were I to enumerate all the cases which I have treated in this way, the number would amount to many hundreds. But you are not to infer that I have cured all the patients I have treated with this remedy, or that I have been always as fortunate as in the cases here reported. I have brought these forward to show the value of the stream of warm water, and not to claim for it infallibility. Like all other remedies, it must fail occasionally; but it has done more in my hands to subdue local inflammation than any other remedy, and I trust it will do as much in your practice. Some of you have seen it used in a few cases, and thus your own observation has taught you to estimate it; but when you have seen more of it, you will then be fully satisfied that I have not insisted too strongly upon its value.

